# Supplementary material for: Cbfβ regulates Wnt/β-catenin, Hippo/Yap, and TGFβ signaling pathways in articular cartilage homeostasis and protects from ACLT surgery-induced osteoarthritis
Source: bioRxiv. 2024 Jan 16:2024.01.15.575763. Preprint. [Version 1] doi: 10.1101/2024.01.15.575763 (PMC10827176; doi:10.1101/2024.01.15.575763)
Supplement: Supplement 1 [file NIHPP2024.01.15.575763v1-supplement-1.pdf]

bioRxiv preprint doi: https://doi.org/10.1101/2024.01.15.575763; this version posted January 16, 2024. The copyright holder for this preprint (which was not certified by peer review) is the author/funder, who has granted bioRxiv a license to display the preprint in perpetuity. It is made available under aCC-BY 4.0 International license.

Cbfβ regulates Wnt/β-catenin, Hippo/Yap, and TGFβ signaling pathways in articular cartilage homeostasis and protects from ACLT surgery-induced osteoarthritis.

Wei Chen[1, *], Yun Lu[2], Yan Zhang[2], Jinjin Wu[2], Abigail McVicar[1], Yilin Chen[1], Siyu Zhu[1], Guochun Zhu[2], You Lu[1], Jiayang Zhang[1], Matthew McConnell[1], and Yi-Ping Li[1, *]

SUPPLEMENTAL FIGURES AND FIGURE LEGENDS

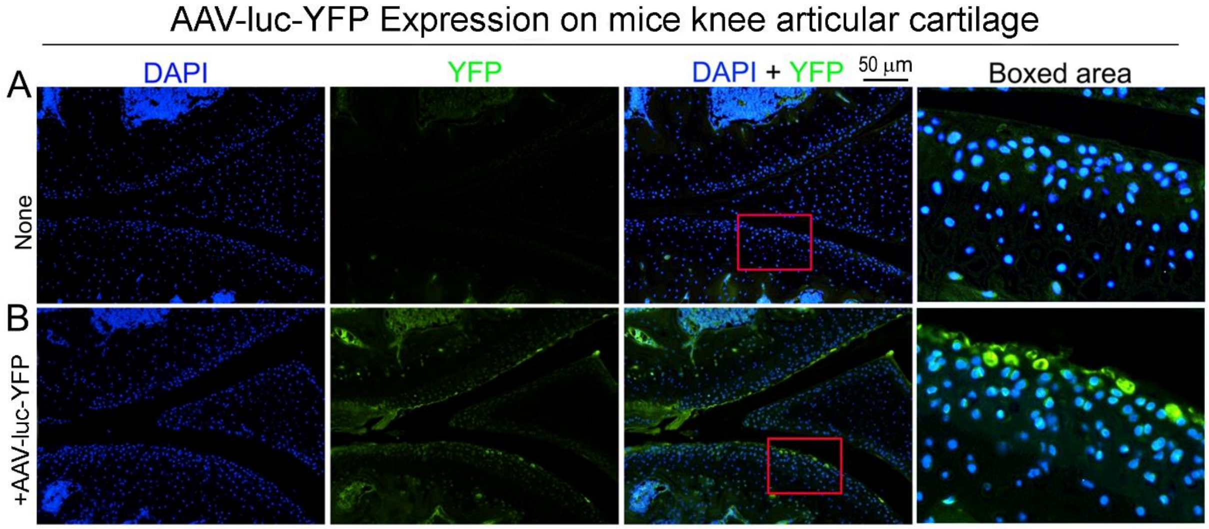

Supplementary Figure 1. Successful AAV-luc-YFP infection in mice Observed by fluorescence microscope**. (A)** DAPI staining for 8-weeks-old male mouse knee articular cartilage Observed by fluorescence microscope. **(B)** DAPI staining and expression for YFP in 8-weeks-old male mice knee articular cartilage Observed by fluorescence microscope. Scale bar: 50 μm (A, B). (n=3)

bioRxiv preprint doi: https://doi.org/10.1101/2024.01.15.575763; this version posted January 16, 2024. The copyright holder for this preprint (which was not certified by peer review) is the author/funder, who has granted bioRxiv a license to display the preprint in perpetuity. It is made available under aCC-BY 4.0 International license.

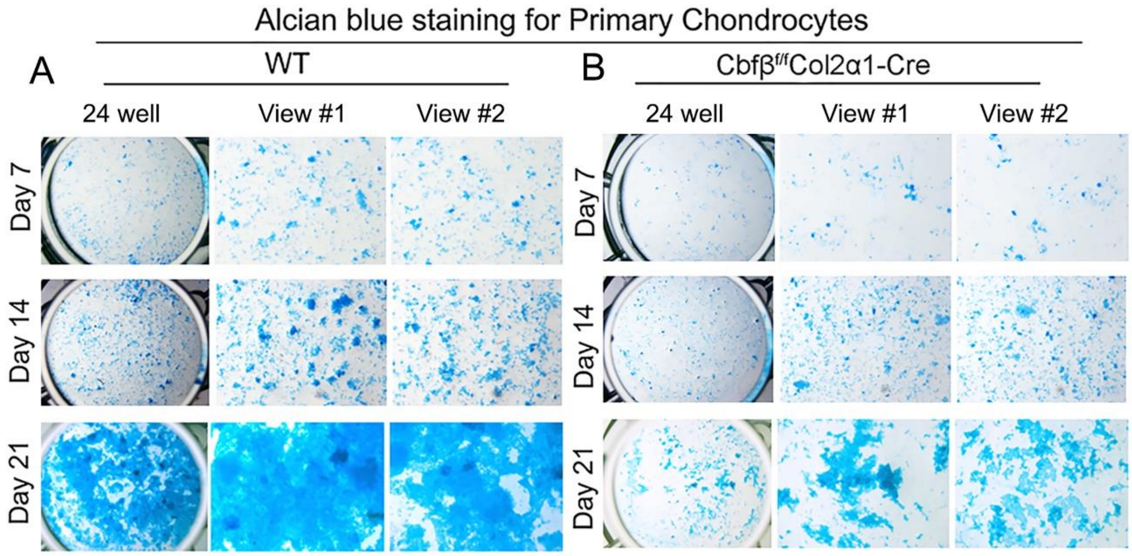

Supplementary Figure 2. Alcian Blue staining of primary chondrocytes from *Cbfβ* deficient newborn mice show reduced matrix deposition. (A) Alcian Blue staining of newborn WT mouse primary chondrocytes. **(B)** Alcian Blue staining of newborn (P0) *Cbfβ$^{f/f}$Col2α1-Cre* mouse primary chondrocytes. (n=5)
